# Temporal dynamics of *Candida albicans* morphogenesis and gene expression reveals distinctions between *in vitro* and *in vivo* filamentation

**DOI:** 10.1128/msphere.00110-24

**Published:** 2024-03-19

**Authors:** Rohan S. Wakade, Melanie Wellington, Damian J. Krysan

**Affiliations:** 1 Department of Pediatrics, Carver College of Medicine, University of Iowa, Iowa City, Iowa, USA; 2 Department of Molecular Physiology and Biophysics, Carver College of Medicine, University of Iowa, Iowa City, Iowa, USA; University of Michigan, Michigan Medicine, Ann Arbor, Michigan, USA

**Keywords:** *Candida albicans*, hyphal development, morphogenesis

## Abstract

*Candida albicans* is a common human fungal pathogen that is also a commensal of the oral cavity and gastrointestinal tract. *C. albicans* pathogenesis is linked to its transition from budding yeast to filamentous morphologies including hyphae and pseudohyphae. The centrality of this virulence trait to *C. albicans* pathobiology has resulted in extensive characterization of a wide range of factors associated with filamentation with a strong focus on transcriptional regulation. The vast majority of these experiments have used *in vitro* conditions to induce the yeast-to-filament transition. Taking advantage of *in vivo* approaches to quantitatively characterize both morphology and gene expression during filamentation during mammalian infection, we have investigated the dynamics of these two aspects of filamentation *in vivo* and compared them to *in vitro* filament induction with “host-like” tissue culture media supplemented with serum at mammalian body temperature. Although filamentation shares many common features in the two conditions, we have found two significant differences. First, alternative carbon metabolism genes are expressed early during *in vitro* filamentation and late *in vivo*, suggesting significant differences in glucose availability. Second, *C. albicans* begins a hyphae-to-yeast transition after 4-h incubation while we find little evidence of hyphae-to-yeast transition *in vivo* up to 24 h post-infection. We show that the low rate of *in vivo* hyphae-to-yeast transition is likely due to the very low expression of *PES1*, a key driver of lateral yeast *in vitro* and that heterologous expression of *PES1* is sufficient to trigger lateral yeast formation *in vivo*.

**IMPORTANCE:**

*Candida albicans* filamentation is correlated with virulence and is an intensively studied aspect of *C. albicans* biology. The vast majority of studies on *C. albicans* filamentation are based on *in vitro* induction of hyphae and pseudohyphae. Here we used an *in vivo* filamentation assay and *in vivo* expression profiling to compare the tempo of morphogenesis and gene expression between *in vitro* and *in vivo* filamentation. Although the hyphal gene expression profile is induced rapidly in both conditions, it remains stably expressed over a 12-h time course *in vivo* while it peaks after 4 h *in vitro* and is reduced. This reduced hyphal gene expression *in vitro* correlates with reduced hyphae and increased hyphae-to-yeast transition. By contrast, there is little evidence of hyphae-to-yeast transition *in vivo*.

## INTRODUCTION

*Candida albicans* is a component of the human mycobiome that also causes disease in both immunocompetent and immunocompromised patients (1, 2). The transition of *C. albicans* from harmless commensal to invasive pathogen is associated with a morphological switch from budding yeast to filaments comprised of both hyphae and pseudohyphae (3). In general, *C. albicans* strains and mutants that show low rates of filamentation are more fit in the commensal setting and less fit during invasive infection (4, 5). Over the years, filamentation has been one of the most intensively studied *C. albicans* virulence traits (3). The vast majority of these studies used one of a number of *in vitro* conditions to generate filaments. Recently, we developed an *in vivo* imaging approach for the analysis of *C. albicans* filamentation during infection. In this approach, *C. albicans* is inoculated directly into the subdermal ear tissue of the mouse (6). Anatomically, this compartment is stromal tissue beneath an epithelium, in this case skin epithelium, and shares many features with stromal tissue beneath the colonized epithelium of the mouth and GI tract. We have used this approach to identify the transcription factor network that regulates filamentation *in vivo* (7) as well as to characterize the filamentation of clinical isolates and protein kinase mutants (8, 9). These studies have revealed a number of important differences in the genes required for filamentation *in vivo* compared to *in vitro*. For example, the cAMP-Protein Kinase A pathway is absolutely required for *in vitro* filamentation in all inducing media but is dispensable for filamentation *in vivo* (9).

The first goal of this work was to characterize the temporal dynamics of filamentous morphogenesis and associated gene expression *in vivo* beginning at early time points using the imaging assay. We coupled this with *in vivo* expression analysis using Nanostring nCounter technology to profile the expression of a set of 186 environmentally responsive genes over the same time periods (7, 9). This set of genes includes 57 (30%) hyphae-associated genes (7). Our previous Nanostring-based *in vivo* expression profiling of *C. albicans* infection of ear tissue, kidney tissue, and oral tissue at single time points has shown both similarities and differences within these niches (9). Furthermore, expression profiles of *in vitro* hyphal induction also show similarities and differences to *in vivo* expression.

To further explore the similarities and differences between filamentation under *in vivo* and *in vitro* conditions, we followed morphogenesis and gene expression over an identical 12-h time course of *in vitro* and *in vivo* filamentation. Nanostring nCounter technology was used to analyze gene expression for two primary reasons. First, genome-wide RNA-seq methods for the direct analysis of *C. albicans* gene expression in infected tissue have not been developed. Although a gene-enrichment strategy has been reported (10), its application to time course analysis was cost prohibitive. Second, we were most interested in the temporal dynamics of a set of well-studied hyphae-associated environmentally responsive genes; therefore, genome-wide characterization of expression was not necessary for our purposes (11). As such, this approach limits the conclusions we can make about global patterns of gene expression.

A single *in vitro* filament-inducing condition was used and was chosen because it is generally used in the field to approximate a host-like environment. Specifically, the tissue culture medium RPMI 1640 supplemented with 10% bovine calf serum (BCS) was used and incubations were performed at mammalian body temperature (37°C). Previous microarray-based expression profiling of the time course for *in vitro C. albicans* filamentation used rich medium (YPD or YEPD) supplemented with 10% BCS (12). It has become well established that the specific filament-inducing medium has a significant effect on both the regulatory pathways and gene expression patterns involved in *C. albicans* filamentation (13). Indeed, those considerations motivated our study of gene expression patterns *in vivo*.

To date, we have performed hundreds of *in vivo* imaging assays with WT cells, the majority of which examined filamentation at 24 h. *In vitro*, hypha begins to form lateral yeast cells after ~4 h of induction; however, we have rarely observed lateral yeast cell formation *in vivo*. Therefore, the second goal of this study was to explore the mechanistic basis underlying this distinction between *in vitro* and *in vivo* morphogenesis. As detailed below, our data suggest that *C. albicans* begin a hyphae-to-yeast transition that is correlated with a reduction in the expression of hyphae-associated genes after ~4-h induction (14) and that this is likely to involve *PES1*, a gene known to drive lateral yeast formation *in vitro* (15). *In vivo*, *C. albicans* cells maintain expression of hyphae-associated dreams and have very low expression of *PES1* throughout the time course, a result that explains why we find little evidence of the hyphae-to-yeast transition *in vivo*. Consistent with this model, we show that expression of *PES1* from a strong, heterologous promoter is sufficient to drive lateral yeast formation *in vivo*.

## RESULTS

### *In vivo* filamentation is initiated rapidly and reaches a steady state 12 h after infection

**Fig 1:**
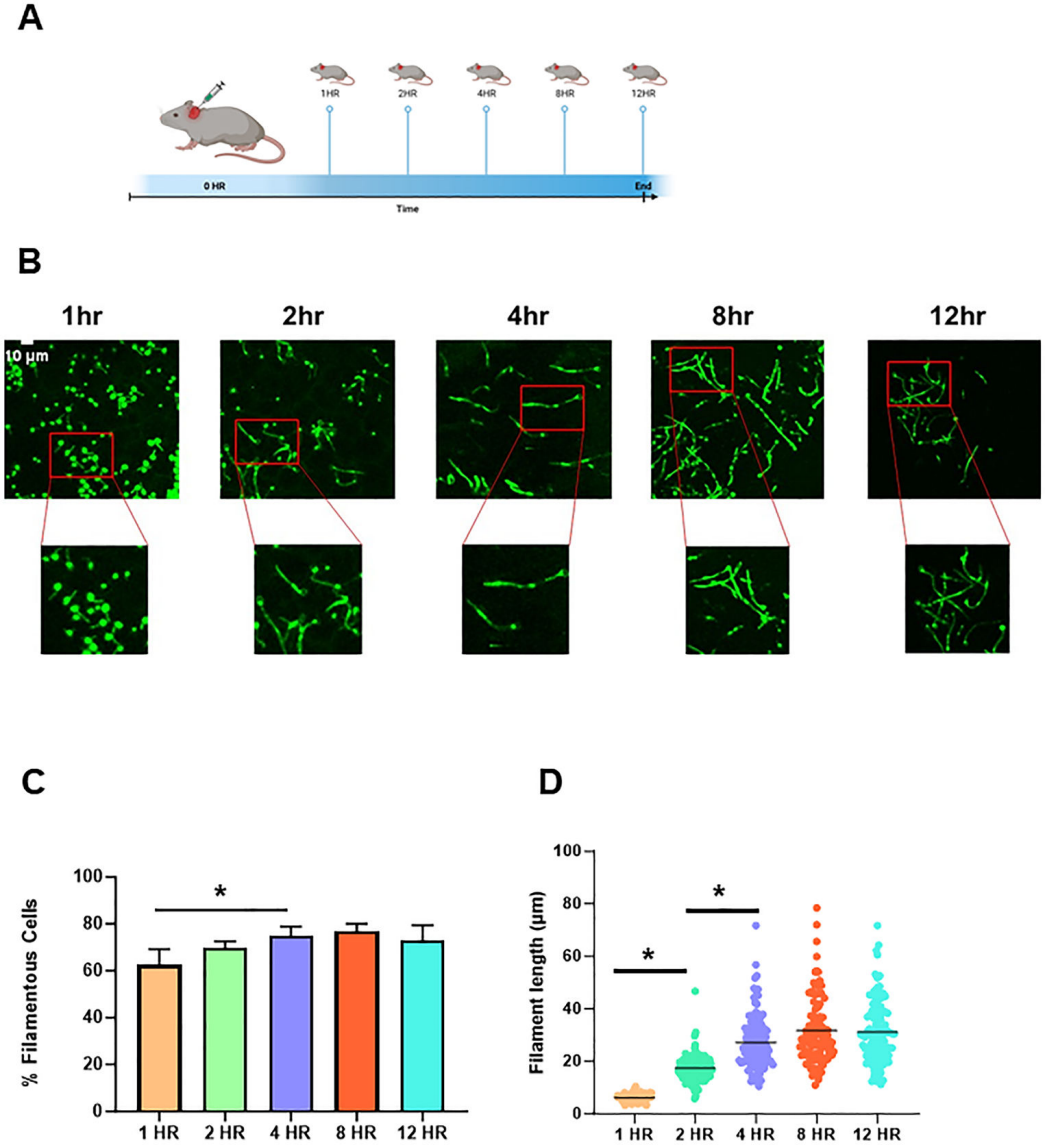
Time course of *in vivo C. albicans* filamentation. (A) Diagram of time course experiment. (**B) **Representative 2D fields from confocal microscopy of NEON-labeled SN250 strain in ear tissue at the indicated time points after infection. (**C) **Quantification of percent filamentous cells at the indicated time points. Asterisks indicated significant differences between time points (*P* < 0.05 *, ANOVA with Tukey’s method of multiple comparison correction). (**D) **The length of filaments at the indicated time points. Asterisks indicate significant differences between the groups (*P* < 0.05 *, Mann-Whitney test).

Stationary phase *C. albicans* SN250 labeled with NEON were injected into the subdermal tissue of mice ears and imaged at 1 h, 2 h, 4 h, 8 h, and 12 h post-infection (Fig. 1A). Germ tube-like cells with short filaments are initially observed in 60% of cells 1 h post-infection (Fig. 1B and C). The percentage of cells that have a filamentous morphology increased slightly until 4 h at which a steady-state ratio of filamentous cells to yeast cells was reached. Previously reported data at 24 h indicate that there is little change in this ratio between 12 h and 24 h. The length of the filaments increases over the first few hours of the time course (Fig. 1D) and reaches a steady state at 8 h. The median length remains constant between 8 h and 12 h and comparison to previously reported data for 24 h indicates that the median length of the population remains relatively stable to that time point.

**Fig 2:**
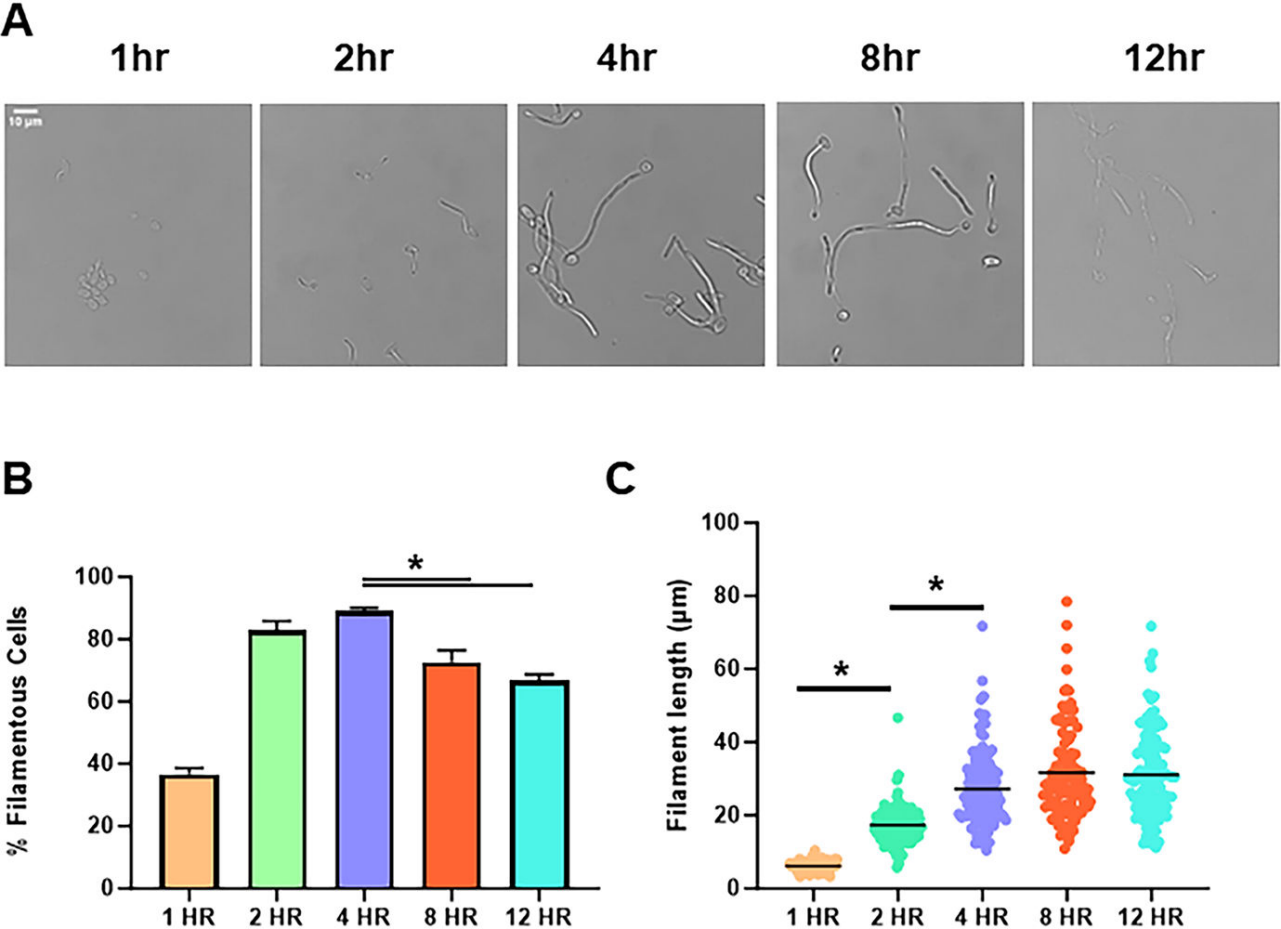
Time course of *in vivo C. albicans* filamentation. (A) Representative bright field images of cell morphology following hyphal induction in RPMI tissue culture medium supplemented with 10% bovine calf serum at 37°C for the indicated times. (**B) **Quantification of percent filamentous cells at the indicated time points. The asterisk indicated significant differences between time points (*P* < 0.05 *, ANOVA with Tukey’s method of multiple comparison correction). (**C)** The length of filaments at the indicated time points. Asterisks indicate significant differences between the groups (*P* < 0.05 *, Mann-Whitney test).

The same time course experiment was conducted for *in vitro* filamentation by inoculating stationary phase cells into RPMI 1640 with 10% BCS at 37°C and fixing cells at each time point. As expected, germ tubes are also initially observed *in vitro* after 1 h (Fig. 2A and B) but the proportion of cells that have formed a germ tube is significantly less *in vitro* relative to *in vivo* conditions after 1 h (60% *in vivo* and 20% *in vitro*). The percentage of filamentous cells peaks at 4 h. In contrast to *in vivo* conditions, the percentage of filamentous cells then declines by ~25% between 4 h and 12 h (*P* < 0.05). Similar to *in vivo* filamentation, the median length of the filaments increases over the first 4 h to a steady state (Fig. 2C). These data indicate that *in vitro* filamentation in RPMIS is slightly delayed relative to *in vivo* conditions and that the ratio of filaments to yeast begins to decline after a peak at 4 hr.

### Expression profiles of *in vivo* and *in vitro* filaments evolve over the time course

**Fig 3:**
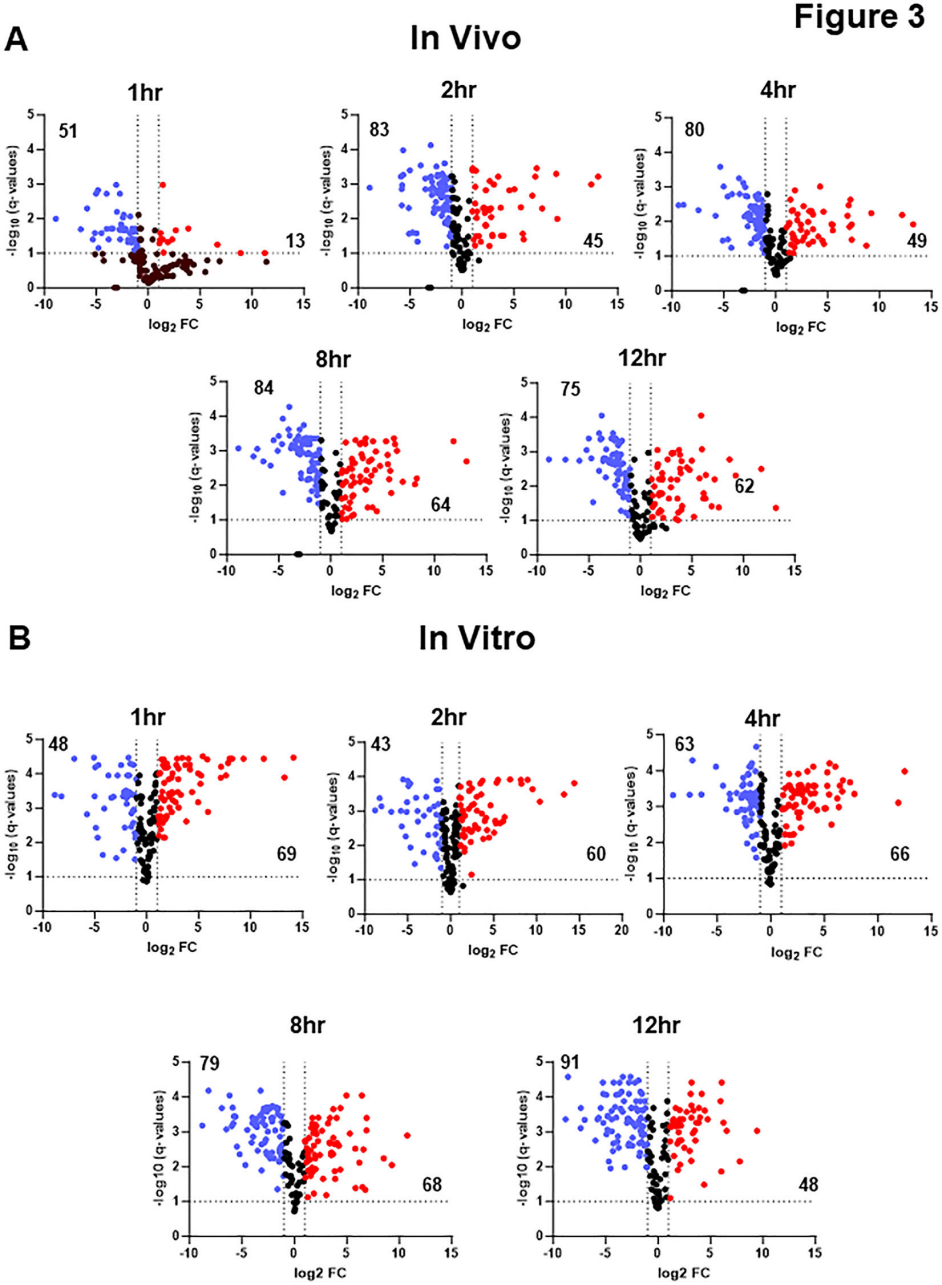
Nanostring analysis of gene expression over time for *in vivo* and *in vitro* filamentation. Volcano plots showing genes with significant (log_2_ ±1; FDR < 0.1, Benjamini method) increase (red dots) or decrease (blue dots) at the indicated time points for *in vivo* filamentation (**A**) and *in vitro* filamentation (**B**). Expression is normalized to yeast phase cells used to infect mice or inoculate *in vitro* cultures. The numbers in the two quadrants indicate the total number of differentially expressed genes for that region of the plot.

**Fig 4:**
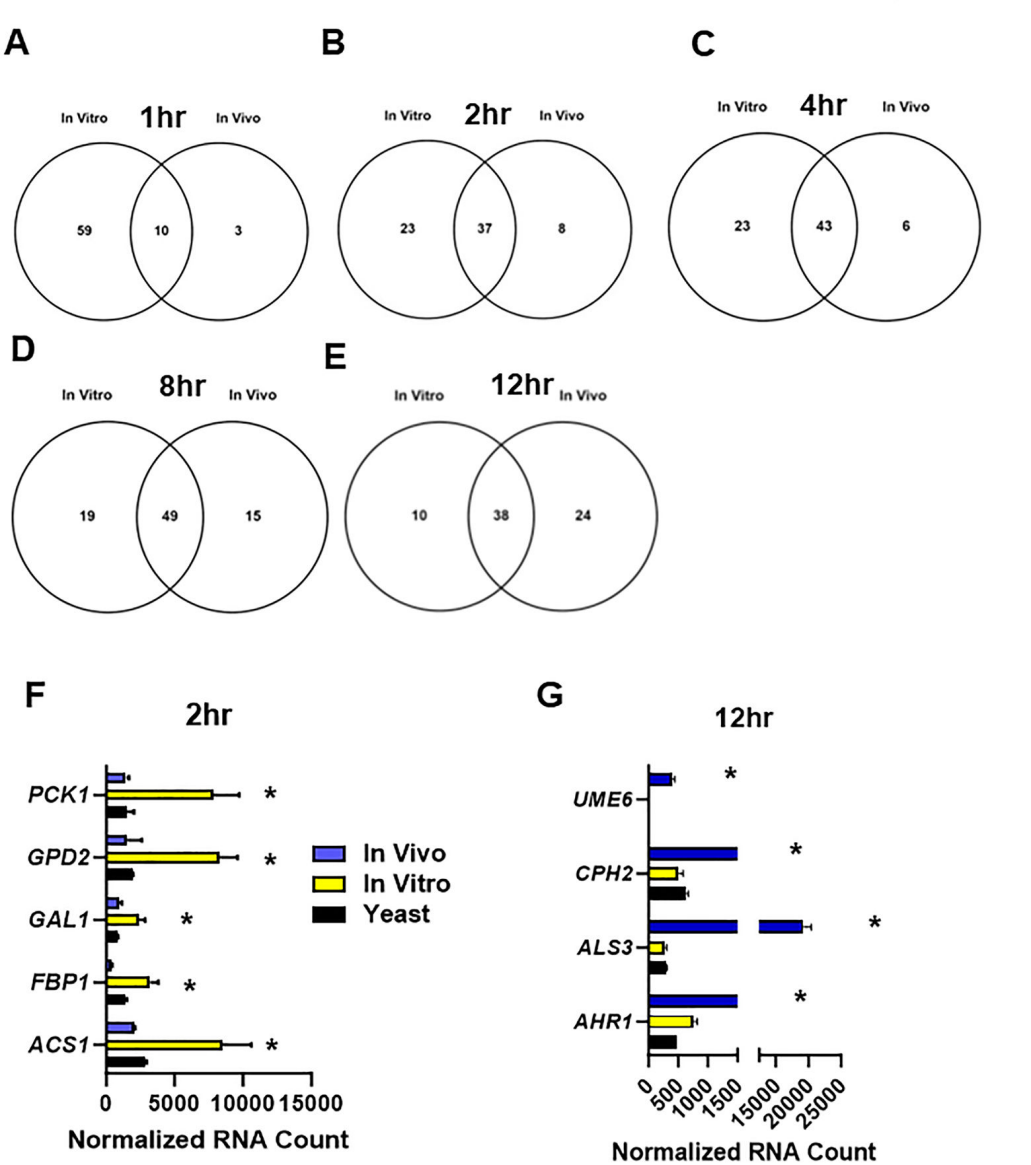
Distinct patterns of upregulated genes during *in vitro* and *in vivo* filamentation. Venn diagrams comparing the profiles of upregulated genes *in vitro* and *in vivo* at 1 h (**A**); 2 h (**B**); 4 h (**C**); 8 h (**D**); and 12 h (**E**). **(F)** Set of alternative carbon metabolism genes upregulated at the 2-h time point during *in vitro* induction relative to yeast inoculum (* indicates FDR < 0.1) but not significantly different from inoculum *in vivo*. (**G)** Set of hypha-associated genes upregulated *in vivo* at the 12-h time point relative to yeast inoculum (* indicates FDR < 0.1) but not significantly different from inoculum *in vitro*. Bars indicate normalized mRNA counts from three independent experiments with error bars indicating standard deviation.

**Fig 5:**
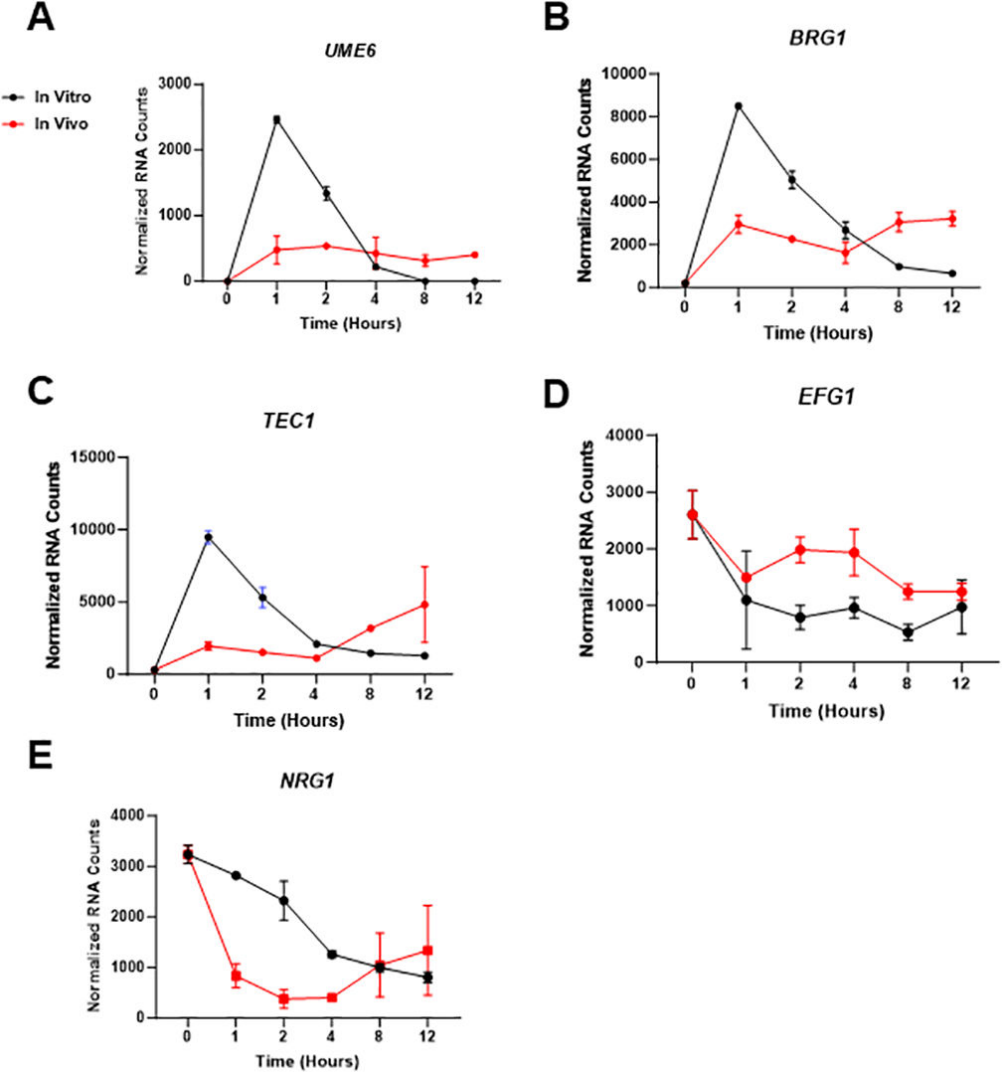
Comparison the dynamics of expression for hyphae-associated transcriptional regulators during *in vitro* and *in vivo* filamentation. (A) Normalized mRNA counts for the expression of *UME6* (**A**), *BRG1* (**B**), *TEC1* (**C**), *EFG1* (**D**), and *NRG1* (**E**) at the indicated time points. Bars indicate normalized mRNA counts from three independent experiments with error bars indicating standard deviation.

**Fig 6:**
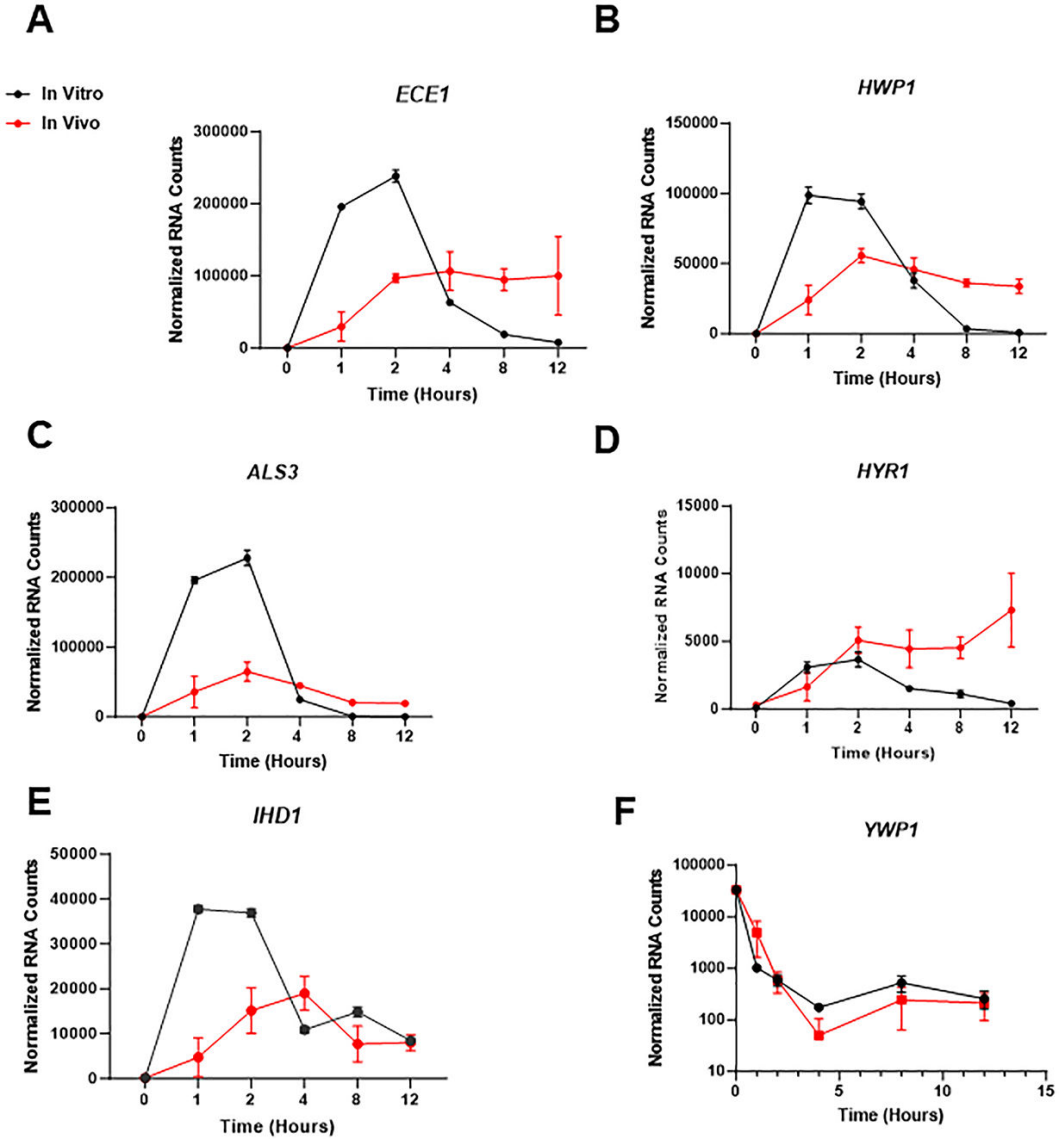
Comparison the dynamics of expression for hyphae- and yeast-associated genes during *in vitro* and *in vivo* filamentation. (A) Normalized mRNA counts for the expression of *ECE1* (**A**), *HWP1* (**B**), *ALS3* (**C**), *HYR1* (**D**), *IHD1* (**E**), and *YWP1* (**F**) at the indicated time points. Bars indicate normalized mRNA counts from three independent experiments with error bars indicating standard deviation.

Nanostring nCounter methods were used to characterize the expression of 186 genes and compared them to the yeast inoculum at 1, 2, 4, 8, and 12 h post-infection (Table S1) as well as post-filament induction *in vitro* (Table S2). The raw, normalized, and processed data are provided in Tables S1 and S2 for each time point. Differentially expressed genes were defined as those with ±2-fold change in expression with an FDR < 0.1 as determined by the Benjamini-Yeuketil procedure when compared to expression in the yeast phase inoculum which was grown overnight at 30°C in rich medium (yeast peptone with 2% dextrose, YPD). The time course of transcriptional changes is summarized by volcano plots in Fig. 3A and B for *in vivo* and *in vitro* conditions, respectively. The total number of differentially expressed genes at each time point is indicated in Fig. 3A and B. We generated Venn diagrams to compare the genes upregulated under *in vitro* conditions to *in vivo* at each time point as a way to assess the similarity and differences between the two conditions at the same time point (Fig. 4A through E). At 1 h, the set of genes induced by a statistically significant amount is low because of relatively high variability (Table S1); this variability resolves by 2 h. As expected, the set of upregulated genes common to both *in vivo* and *in vitro* conditions at 1 h includes regulators of hyphae morphogenesis (*BRG1*, *CPH1/2*, *TEC1*, and *UME6*) as well as hyphae-associated cell wall genes (*ALS1* & *HWP1*); see Table S1 graphs (Fig. 5 and 6).

At early time points (Fig. 4A through C), the majority of genes upregulated *in vivo* are also upregulated *in vitro*. However, a large proportion of genes are uniquely upregulated *in vitro*. For example, one-third of the genes upregulated *in vitro* at 2 h were not upregulated *in vivo*. The set uniquely upregulated *in vitro* is enriched for carbon metabolism genes (FDR 0.01, Benjamini-Yeuktiel: *PCK1*, *GPD2*, *GAL1*, *ACS1*, *FBP1*, Fig. 4F); *PCK1* and *FBP1* are involved in gluconeogenesis and *ACS1* is induced by low glucose (16), suggesting that glucose availability may be distinct between *in vitro* and *in vivo* conditions at early time points. Further supporting that possibility, *AOX2*, which is induced by low glucose conditions (17), was strongly upregulated at 1 h (59-fold increase relative to yeast, FDR = 0.005) and 2 h (22-fold increase relative to yeast, FDR = 0.006) time points *in vitro*, while *AOX2* expression is below the level of detection *in vivo* (Table S1). At later time points *in vivo*, *AOX2* (12 h, 69-fold increase relative to yeast, FDR = 0.02) and *PCK1* were upregulated (12 h, fourfold increase relative to yeast, FDR = 0.05), suggesting that the cells begin to depend more on non-glucose carbon sources as the time course progresses.

At the 12-h time point, more genes are uniquely upregulated *in vivo* compared to *in vitro* (Fig. 4E). The set of 24 genes upregulated only *in vivo* at the 12-h time point is enriched for zinc (FDR: 2e^−4^; *ZAP1*, *ZRT1,* and *PRA1*) and iron (FDR: 2e^−3^; *HAP3*, *FTR1*, *RBT5*, and *PGA7*) homeostasis (18, 19). Interestingly, filamentous growth-associated genes are also enriched in this set (FDR: 3e^−4^) including transcriptional regulators of filamentous growth *UME6*, *AHR1*, and *CPH2* and the hyphae-associated cell wall gene *ALS3* (Fig. 4G). We were particularly struck by the fact that *UME6* expression was no longer upregulated after 12 h of induction *in vitro*. Indeed, *UME6* expression had returned to the near background levels of expression by 8 h of *in vitro* induction and was not different than in the yeast phase (Fig. 5A). *UME6* expression is required for the maintenance of hyphal elongation both *in vitro* and *in vivo* (7, 20). The decline in the expression of *UME6* after 8 h of *in vitro* hyphal induction suggests that the hyphal transcriptional program is reduced late in the time course, whereas *in vivo* the activity of this program appears to be maintained longer.

### The expression of hyphae-induced genes decreases over time *in vitro* and correlates with reduced filaments at those time points

To further evaluate the possibility that the hyphal morphogenic and transcriptional program was waning after 4 h *in vitro* but not *in vivo*, we compared the expression of transcription factors that positively regulate filamentation *in vitro* and *in vivo*. In addition to *UME6*, the hyphae-induced transcription factors (TFs) *BRG1* and *TEC1* are upregulated after 1-h exposure to RPMIS and 1 h post-infection *in vivo* (Fig. 5A through C). *In vitro*, the expression of all three TFs peaks and then falls. By contrast, *in vivo* expression of *UME6*, *BRG1*, and *TEC1* shows a slower slope of initial induction and then maintains relatively stable levels throughout the time course. The hyphae-associated TFs *CPH1* and *CPH2* also follow the same pattern of expression but are not as strongly induced (Tables S1 and S2). *EFG1* is a critical regulator of the hyphal transcriptional profile under many conditions (21). Interestingly, and in contrast to the other three TFs, *EFG1* expression is reduced relative to the yeast phase both *in vitro* and *in vivo* (Fig. 5D). Finally, expression of the repressor of filamentation *NRG1* (22, 23) is also downregulated under both conditions, although this downregulation occurs slightly more rapidly *in vivo* (Fig. 5E).

Consistent with the temporal pattern for the expression of hyphae-induced TFs *in vitro*, the expression of hyphae-associated target genes *ALS3*, *ECE1*, *HWP1*, *HYR1,* and *IHD1* show the same peak and decline over time. Once again, the tempo for the expression of these genes *in vivo* is distinct with a more gradual increase followed by relatively stable expression over the time course (Fig. 6A through E). We also examined the expression of the yeast-specific cell wall protein *YWP1* (24) over the time course to see whether the reduced expression of hyphae-associated genes was accompanied by a corresponding increase in the expression of a yeast-associated gene. As shown in Fig. 6F, the expression of *YWP1* declines in both *in vitro* and *in vivo* conditions and remains low relative to yeast with a slight increase in expression under both conditions after a nadir at 4 h. Thus, despite the reduction in the expression of hyphae-associated TFs and other hyphae-associated gene at later time points *in vitro*, expression of the *YWP1* remains low at those same time points relative to yeast phase cells. Overall, the decreased expression of hyphae-associated genes at later time points correlates with the decrease in the proportion of filaments after a peak at approximately 4 h. The expression of the same genes *in vivo* is stable over the same time period and also correlates with the stable extent of filamentation over the same time points *in vivo*.

### Over a 24-h time course *in vitro* filaments generate lateral yeast but *in vivo* filaments do not

**Fig 7:**
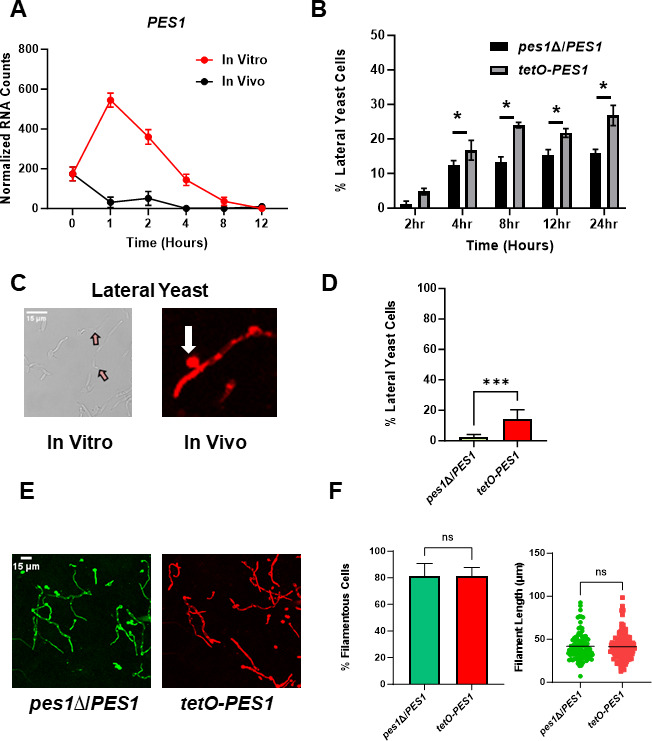
Heterologous expression of *PES1* is sufficient to induce lateral yeast formation *in vivo*. (A) Normalized mRNA counts for the expression of *PES1*. Bars indicate normalized mRNA counts from three independent experiments with error bars indicating standard deviation. (**B)** Effect of heterologous expression of *PES1* on lateral yeast formation over time course of *in vitro* hyphal induction. Bars indicate the percentage of filaments with lateral yeast cells. * indicates statistically significant differences between groups (two-way ANOVA corrected for multiple comparisons with Sidak’s test). (**C)** Representative images of lateral yeast *in vitro* and *in vivo*. (**D)** Lateral yeast formation *in vivo* for *pes1*∆/*PES1* and *tetO-PES1* 24 h post-infection. Bars indicate means from two independent experiments with standard deviation indicated by error bars. **** indicates *P* < 0.0001 by student’s *t*-test. (**E) **Representative images of the *pes1*∆/*PES1* and *tetO-PES1* mutant morphologies *in vivo*. (**F)** Quantification of % filamentous cells and filament length for the *pes1*∆/*PES1* and *tetO-PES1* mutant strains. NS indicates no significant difference by Student’s *t*-test (% filamentous cells) and Mann-Whitney test (filament length).

*In vitro*, yeast cells begin to emerge at subapical cell bodies in a process termed the hyphae-to-yeast transition. In our previous large-scale screen comparing *in vivo* filamentation between TF mutants and WT in one-to-one competition experiments (over 150 replicates of WT, (7)), we observed very few lateral yeasts on *in vivo* filaments at the 24-h time point. Although the hyphae-to-yeast transition remains a relatively understudied aspect of *C. albicans* morphogenesis (14), lateral yeast formation is linked to the expression of *PES1* (15). Pes1 is a pescadillo protein that is required for yeast growth and lateral yeast formation as well as biofilm dispersion (25). We, therefore, compared the expression of *PES1* during *in vitro* and *in vivo* filamentation. *In vivo*, *PES1* expression drops dramatically to below or at the background for the majority of the time course (Fig. 7A). By contrast, *PES1* expression initially increases during *in vitro* induction and then falls to essentially background by 12 h post-induction. As reported by Shen et al., over-expression of *PES1* from the *TET*- promoter leads to increased lateral yeast cell formation during a variety of *in vitro* hyphae induction conditions (15). We, therefore, tested whether this was the case for RPMIS using the same strain generated by Shen et al (15); because previous work indicated that lateral yeast formation tends to increase with time, we extended this experiment to 24 h (14). Consistent with previous reports, the *tetO-PES1* strain generated more lateral yeast than a congenic strain with *PES1* expressed (pWT-*PES1*) from its native promoter at all time points (Fig. 7B).

To determine whether increased expression of *PES1* was sufficient to drive lateral yeast formation *in vivo*, we labeled the *tetO-PES1* and p*WT-PES1* strains with NEON and iRFP, respectively, and inoculated a 1:1 mixture into the ear. In Fig. 7C, we show representative examples of lateral yeast identified *in vivo* which can be compared to hyphal branching which is shown in Fig. S1A. As shown in Fig. 7D, the *tetO-PES1* strain formed dramatically more lateral yeast than the strain expressing *PES1* from the native promoter. This suggests that the low expression of *PES1 in vivo* contributes to the near absence of lateral yeast and that increased *PES1* expression can drive lateral yeast formation *in vivo*. Interestingly, increased expression of *PES1 in vitro* under some conditions reduces hyphae formation and the length of hyphae (15). However, the *tetO-PES1* strain formed hyphae to the same extent with similar lengths as the comparator strain (Fig. 7E and F).

Finally, reduced cAMP-PKA pathway activity has also been shown to increase the hyphae-to-yeast transition *in vitro* (26, 27). We found that mutants lacking the PKA pathway components Cyr1 and Tpk1/2 can form filaments *in vivo* (9). We, therefore, examined these strains for *in vivo* lateral yeast formation but found no significant difference relative to control strains (Fig. S1B and C). For these experiments, we compared the *tetO-PES1/pes1∆* strains to the corresponding *pes1*∆/*PES1* heterozygous mutant. Therefore, it is possible that the heterozygous *pes1*∆/*PES1* has lower lateral yeast formation due to haploinsufficiency (15). However, the lateral yeast formed by the *pes1*∆/*PES1* mutant is not different from those formed by an SC5314-derived reference strain with both alleles of *PES1* (Fig. S1C). Taken together, our data indicate that in the first 24 h of filamentation *in vivo* lateral yeast formation is extremely rare and that low *PES1* expression throughout the course of filamentation is likely to be a contributing factor.

## DISCUSSION

Hyphal morphogenesis remains one of the most intensely studied aspects of *C. albicans* pathobiology (3). Most of this work has taken advantage of the wide range of *in vitro* media and conditions that induce the transition of *C. albicans* from a budding yeast morphology to the filamentous hyphal and pseudohyphae. We have begun to characterize this transition in mammalian tissue through the use of an *in vivo* imaging approach (6–9). Here, we compared the time dependence of *in vivo* filamentation and in the setting of a commonly used “host-like” induction condition (RPMI +10% BCS at 37°C). Although we have found some significant differences between *in vivo* and *in vitro* conditions, it is important to first point out the similarities, particularly during early time points. The rapid induction of positive regulators and repression of the major repressor of filamentation is observed under both conditions. By 2 h, there is significant overlap in the transcriptional responses based on a set of genes selected to include hyphae-related genes and environmentally responsive genes.

Generally speaking, recent studies have emphasized the fact that many features of hyphal morphogenesis are dependent upon the specific conditions used to induce that transformation (13). In the two conditions we studied, it appears that glucose availability is low *in vitro*. This conclusion is based on the rapid increase in the expression of genes related to gluconeogenesis and alternative carbon metabolism *in vitro* but not *in vivo*. One possibility for this difference is that *in vitro* cultures have a fixed amount of glucose and other nutrients; once the fixed amount of glucose is depleted, the cells must switch to alternative carbon source metabolism. *In vivo*, *C. albicans* is another organism that delivers glucose and nutrients to its tissues and, as a result, delivers nutrients to *C. albicans*. After 12 h, genes such as that for alternative oxidase, *AOX2*, are beginning to be expressed at high levels, suggesting that the organism is experiencing reduced glucose delivery. This may be due to the beginning of abscess formation or tissue damage and necrosis, leading to disrupted blood flow in the region.

The most striking difference between these two conditions is that the expression of positive transcriptional regulators of hyphae morphogenesis and their targets declines sharply *in vitro* after a peak at the 4-h time point. This reduction in hyphae-associated gene expression correlates with a reduction in the filament-to-yeast ratio between 4 h and 12 h *in vitro*. The reduction in the filament-to-yeast ratio *in vitro* also correlates with the appearance of lateral yeast and follows a peak in the expression of the positive regulator of lateral yeast cell development, *PES1*. These observations suggest that the population of cells begins to express features of the hyphae-to-yeast transition after ~4-h induction. In biofilm conditions, the lateral yeast cells lead to the dispersion of yeast cells into the media and we suggest that the increase in lateral yeast cells between 2 h and 4 h and the increase in planktonic yeast cells after 4 h may both be due to the hyphae-to-yeast transition (15, 25). Finally, the increase in lateral yeast formation in a strain that overexpresses *PES1 in vitro* further supports the conclusion that this process is governed, at least in part, by this protein.

*In vivo*, we observed very little evidence of the hyphae-to-yeast transition during the time course examined. Furthermore, we have observed very few lateral yeasts *in vivo* up to 24 h post-infection and the filament-to-yeast ratios are stable over the same time period (7). Once again, these morphological observations can be correlated with the expression patterns of positive regulators of hyphae and the best characterized positive regulator of lateral yeast formation, *PES1*. Specifically, the expression of hyphae-induced transcriptional regulators (*BRG1*, *TEC1*, and *UME6*) increases over the first 1 h and then largely remains stable over the next 12 h and appears to maintain this pattern up to 24 h post-infection based on previously reported data (7). This suggests that, in contrast to *in vitro* hyphae formation, the hyphal transcriptional program remains active throughout the first 12–24 h of *in vivo* infection.

*PES1* expression rapidly drops to near undetectable levels *in vivo* and remains low over the time course. Because lack of *PES1* expression inhibits lateral yeast formation *in vitro* (15, 25), this low expression seems likely to contribute to the low numbers of lateral yeast *in vivo*. As our data indicate, *PES1* expression from a heterologous promoter drives lateral yeast formation *in vivo* at a time point when it does not normally occur. Accordingly, this indicates that *PES1* expression is sufficient to trigger lateral yeast formation *in vivo* and strongly supports the conclusion that low expression of *PES1* is likely to contribute, at least in part, to the low rate of lateral yeast formation *in vivo*. This low level of *PES1* expression is not unique to the ear infection site and we have found similarly low levels of *PES1* expression in both infected kidney and tongue 24 h post-infection (7, 9, 28). Because of its role in virulence, it seems likely that its effects are most important after the initial establishment of infection. Indeed, Uppuluri et al. concluded that the role of *PES1* is most important after the establishment of infection based on the time dependence of its effect on fungal burden (29). Our observation of low expression of *PES1 in vivo* is consistent with their findings and conclusions.

Our apparent inability to find strong evidence for the hyphae-to-yeast transition *in vivo* raises the interesting question: why not? A very simple explanation for the low rate of hypha-to-yeast transition *in vivo* may be that the transition may occur later during infection. The high expression of positive transcriptional regulators indicates that the hyphal transcriptional program is consistently maintained thoughout the 24-h period we studied. As such, it seems very possible that the hyphal program begins to wane later in infection, as it does *in vitro*. Our ability to collect high-resolution morphological data at time points beyond 24 h declines because *C. albicans* begins to form dense micro-abscesses in the ear tissue after 24 h (30).

In addition to providing insights into the dynamics of *in vivo* filamentation, our data have implications for the study of *C. albicans* filamentation *in vitro*. Specifically, both the transcriptional and morphological features of the filamentation program in a given medium are likely to vary considerably over time, particularly in later time points. Therefore, it is important to be sure that the selected time point represents the specific stage of filamentation in which one is interested.

In summary, this work provides insights into the temporal dynamics of filamentation in mammalian tissue, allowing the identification of similarities and differences with widely employed host-like *in vitro* conditions. Our previous work has found that there are significant differences in the sets of TFs (7) and protein kinases (9) that regulate *in vivo* filamentation relative to *in vitro* conditions. Many factors are likely to contribute to the differences in the regulatory factors required for *in vivo* filamentation. Based on this work, it seems likely that some of these differences could be due, at least in part, to the distinct environmental conditions encountered during infection and differences in the tempo of morphogenesis.

## MATERIALS AND METHODS

### Strains and media

The SC5314-derived *C. albicans* reference strain SN250 was used for all experiments except for those involving *PES1* mutant strains. The *pes1*∆/*PES1* and *tetO-PES1* strains (15) were generous gifts of Julia Köhler (Harvard) and are in the SC5314 background. All *C. albicans* strains were precultured overnight in yeast peptone dextrose (YPD) medium at 30°C with shaking. Standard recipes were used to prepare YPD (31). RPMI 1640 medium was supplemented with bovine calf serum (10% vol/vol).

### *In vitro* hyphal induction

For *in vitro* hyphal induction, the *C. albicans* strain was incubated overnight at 30°C in YPD media, harvested, and diluted into RPMI +10% bovine calf serum at a 1:50 ratio and incubated at 37°C. Cells were collected at different time points (e.g., 1 h, 2 h, 4 h, 8 h, and 12 h) and processed for microscopy or RNA isolation as described below.

### *In vitro* characterization of *C. albicans* morphology

Induced cells were fixed with 1% (vol/vol) formaldehyde. Fixed cells were then imaged using the Echo Rebel upright microscope with a 60× objective. The assays were conducted in triplicates on different days to confirm reproducibility.

### *In vivo* characterization of *C. albicans* morphology

These assays were performed as previously described (6). Briefly, 1 × 10^6^ WT *C. albicans* cells were inoculated intradermally in mouse ears (3 mice/time point). Mice (3 mice/time point) were sacrificed at each time point, and ears were harvested. One ear/mouse was immediately submerged into the ice-cold RNA later solution and another ear was used for the imaging. Multiple Z stacks (minimum 20) were acquired and used it to score the yeast vs filamentous ratio. Round and/or budded cells were considered “Yeast,” whereas the cells containing intact mother and filamentous, which was at least twice the length of the mother body, were considered “filamentous.” Lateral yeast cells were distinguished from branching hyphae by requiring lateral yeast cells to be no more than two cell body lengths long and have curved than parallel cell walls (See Fig. 7C; Fig. S1A). A minimum of 100 cells from multiple fields were scored. Student’s *t*-test was performed to define the statistical significance between the different time points. All animal experiments were approved by the University of Iowa IACUC.

### RNA extraction

*In vitro* and *in vivo* RNA extraction were carried out as described previously (7, 9). For *in vitro* RNA extraction, cells were collected at different time points, centrifuged for 2 min at 10 K rpm at room temperature and RNA was extracted according to the manufacturer’s protocol (MasterPure Yeast RNA Purification Kit). For *in vivo* RNA extraction, mice were euthanized, ear tissue was harvested, and the tissue was placed into the ice-cold RNA-Later solution. Ear tissue was then transferred to the mortar and flash-frozen with liquid nitrogen. Using a pestle, the frozen was ground to a fine powder. The resulting powder was collected and 1 mL of ice-cold Trizol was added. The samples were placed on a rocker at RT for 15 min and then centrifuged at 10K at 4°C for 10 min. The cleared Trizol was collected into a 1.5 mL Eppendorf tube and 200 µL of RNase-free chloroform was added to each sample. The tubes were shaken vigorously for 15 s and kept at RT for 5 min followed by centrifuge at 12 K rpm at 4°C for 15 min. The cleared aqueous layer was then collected into a new 1.5 mL Eppendorf tube and RNA was further extracted following the Qiagen RNeasy kit protocol.

### NanoString gene expression analysis

NanoString analysis was carried out as described previously (7, 9). Briefly, in total, 40 ng of *in vitro* or 1.4 µg of *in vivo* RNA was added to a NanoString codeset mix and incubated at 65^0^ C for 18 h. After the hybridization reaction, samples proceeded to the nCounter prep station and samples were scanned on a nCounter digital analyzer. nCounter .RCC files for each sample were imported into nSolver software to evaluate the quality control metrics. Background subtraction was performed using negative control probes to establish a background threshold, which was then subtracted from the raw counts. The resulting background subtracted total raw RNA counts underwent a two-step normalization process. First normalized against the highest total counts from the biological triplicates and then to the wild-type samples. Differentially expressed genes were defined as those with ±2-fold change in expression with an FDR < 0.1 as determined by the Benjamini-Yeuketil procedure when compared to expression in the yeast phase inoculum which was grown overnight at 30°C in rich medium (yeast peptone with 2% dextrose, YPD).

### Software

Quantitative image analysis was carried out using ImageJ software. GraphPad Prism (V. 9.3.1) was used to plot the graphs and to perform the statistical tests.

## Data Availability

All raw (source) data, normalized data, and large-scale expression data generated by Nanostring are provided in Tables S1 and S2. The bright field and confocal microscopy Z-stacks and images used to characterize *C. albicans* morphology at the different time points are very large files that are not easily deposited or annotated for deposit in public repositories. Therefore, the source data for the imaging figures are available from the corresponding author to interested investigators upon reasonable request.

## SUPPLEMENTAL MATERIAL

Fig. S1 msphere.00110-24-s0001.tif Lateral yeast formation in PKA mutants.

Supplemental Material Summary msphere.00110-24-s0002.docx Legends for all supplemental materials.

Table S1 msphere.00110-24-s0003.xlsx *In vivo* gene expression.

Table S2 msphere.00110-24-s0004.xlsx *In vitro* gene expression.

ASM does not own the copyrights to Supplemental Material that may be linked to, or accessed through, an article. The authors have granted ASM a non-exclusive, world-wide license to publish the Supplemental Material files. Please contact the corresponding author directly for reuse.
