# Supplementary figures and images for: Temporal dynamics of Candida albicans morphogenesis and gene expression reveals distinctions between in vitro and in vivo filamentation
Source: mSphere. 2024 Mar 19;9(4):e00110-24. doi: 10.1128/msphere.00110-24 (PMC11036811; doi:10.1128/msphere.00110-24)

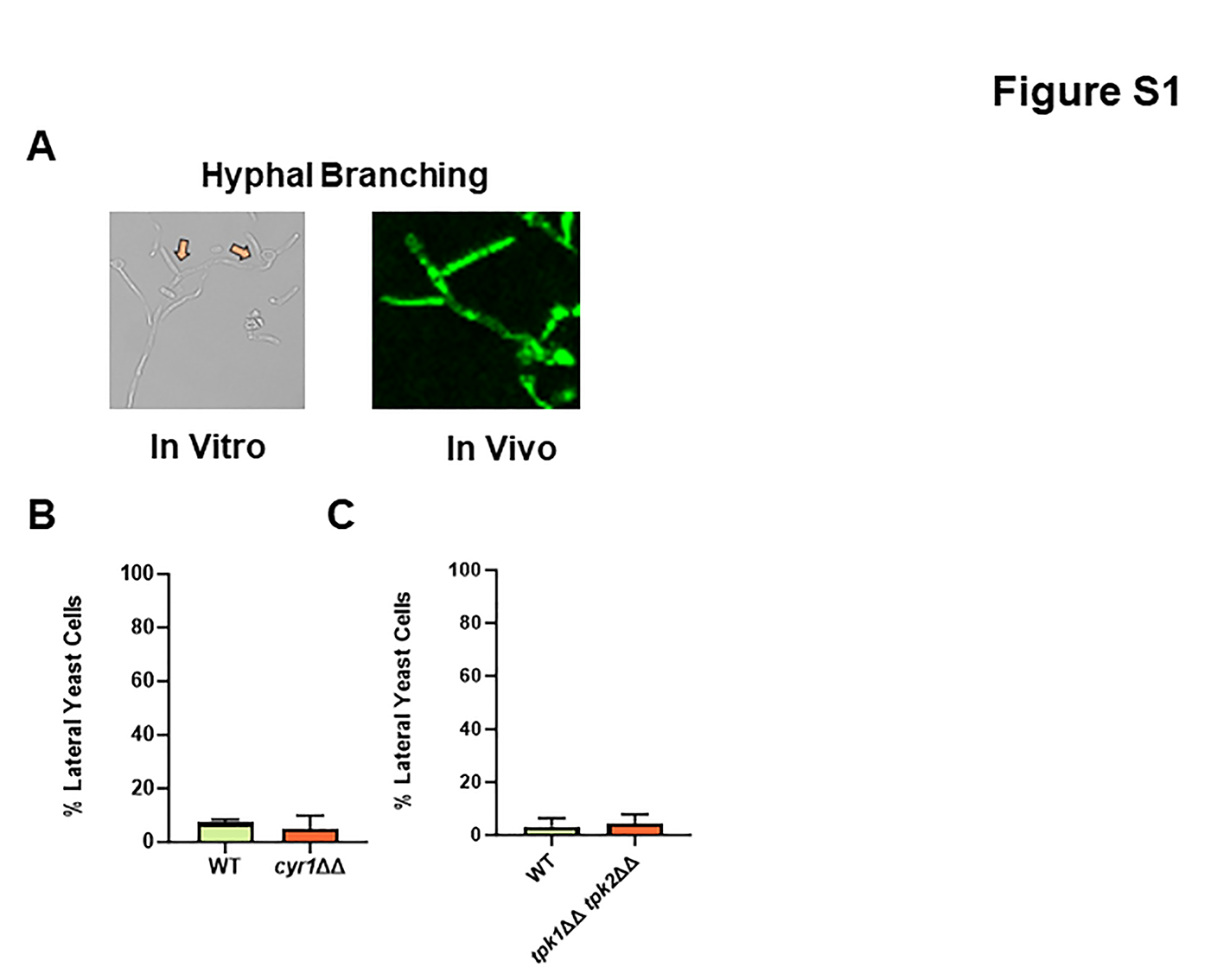

Supplement: Fig. S1 — Lateral yeast formation in PKA mutants. [file msphere.00110-24-s0001.tif]
