# Supplementary material for: Temporal dynamics of Candida albicans morphogenesis and gene expression reveals distinctions between in vitro and in vivo filamentation
Source: mSphere. 2024 Mar 19;9(4):e00110-24. doi: 10.1128/msphere.00110-24 (PMC11036811; doi:10.1128/msphere.00110-24)
Supplement: Supplemental Material Summary — Legends for all supplemental materials. [file msphere.00110-24-s0002.docx]

**Supplementary Materials**

**Table S1.** Nanostring data for in vivo time course with raw counts, background corrected counts, normalized counts, fold change for each gene relative to either yeast phase, Student t-test values, and FDR calculated by the Benjamini-Yekutieli method. Differentially expressed genes were defined as those genes with ± 2-fold change in expression with FDR <0.1. Significantly upregulated genes are indicated by green fold change values at the time point in which they are differentially expressed; red indicates downregulated at that time point.

**Table S2.** Nanostring data for in vitro time course with raw counts, background corrected counts, normalized counts, fold change for each gene relative to yeast phase, Student’s test values, and FDR calculated by the Benjamini-Yekutieli method. Differentially expressed genes were defined as those genes with ± 2-fold change in expression with FDR <0.1. Significantly upregulated genes are indicated by green fold change values at the time point in which they are differentially expressed; red indicates downregulated at that time point.

**Supplementary Figure S1. A**. Representative images of hyphal branching in vitro and in vivo. Lateral yeast formation in vivo for *cyr1*∆∆ (**B**) and *tpk1*∆∆ *tpk2*∆∆ (**C**) mutants 24hr post-infection. Bars indicate means from two independent experiments with standard deviation indicated by error bars. There were no significant differences (p> 0.05) between groups by Student’s t test.
